# Novel Pectin Binder for Satelliting Carbides to H13 Tool Steel for PBF-LB Processing

**DOI:** 10.3390/ma16103649

**Published:** 2023-05-10

**Authors:** Fabian Meyer, Fabian Kolodzy, Marie Luise Scheck, Anke Kaletsch, Tetiana Kharandiuk, Andrij Pich, Christoph Broeckmann

**Affiliations:** 1 DWI—Leibniz Institute for Interactive Materials, Forckenbeckstr. 50, 52056 Aachen, Germany; 2 Institute for Technical and Macromolecular Chemistry, RWTH Aachen University, Worringerweg 2, 52074 Aachen, Germany; 3 Institute of Applied Powder Metallurgy and Ceramics (IAPK) at RWTH Aachen e.V., Augustinerbach 4, 52064 Aachen, Germany; 4 Institute for Materials Applications in Mechanical Engineering, RWTH Aachen University, Augustinerbach 4, 52064 Aachen, Germany; 5 Aachen Maastricht Institute for Biobased Materials (AMIBM), Maastricht University, Brightlands Chemelot Campus, Urmonderbaan 22, 6167 RD Geleen, The Netherlands

**Keywords:** laser-based powder bed fusion, additivation, polymer binder, powder application, microstructure, carbides, tool steel

## Abstract

In order to enhance the range of processable alloys of laser-based powder bed fusion, reinforced alloys have gained focus. Satelliting is a recently introduced method for adding fine additives to larger parent powder particles using a bonding agent. Satellited particles prevent a local demixing due to size and density effects of the powder. In this study, the satelliting method is used for the additivation of Cr_3_C_2_ to AISI H13 tool steel via a functional polymer binder (pectin). The investigation includes a detailed binder analysis and comparison to the previously used PVA binder as well as processability in PBF-LB and the microstructure of the alloy. The results reveal that pectin is a suitable binder for the satelliting process and the demixing behavior that appears when using a simple powder blend can be significantly reduced. However, the alloy is enriched with carbon, which results in austenite being retained. Thus, in future research, a reduced binder content will be investigated.

## 1. Introduction

### 1.1. Motivation

Columnar grain growth originates from crystal growth on an underlying crystal with the same orientation. These grains are commonly observed in laser-based powder bed fusion of metals (PBF-LB/M) [1]. Columnar grain growth during solidification of additively manufactured specimens made of an alloy with a large solidification interval leads to the formation of solidification cracking. Large solidification intervals result from the alloying contents of the material. The solid solution limit of the solidifying phase leads to an enrichment of the melt with alloying elements. As a result, the liquidus temperature of the melt decreases and enlarges the solidification interval. Solidification cracks occur if the residual melt at the grain boundaries solidifies and shrinks and the gaps are not refilled with further melt. In some cases, these cracks are apparent when processing pure AISI H13 [2,3].

A previously published study revealed that modifying AISI H13 with portions of Cr_3_C_2_ can reduce the hot cracking tendency of the alloy due to a narrower solidification interval. The observed microstructure was in good agreement with the results of various other researchers analyzing additively manufactured high alloyed tool steels. The microstructure is commonly reported to contain martensite as well as retained austenite. The retained austenite is enriched, and hence stabilized, by carbide-forming elements. Within the enriched areas, carbides form at the end of solidification or are precipitated during post-processing heat treatments [4,5,6,7,8].

A Cr_3_C_2_ content of 5.4 wt.% in the material showed the shortest hot crack lengths. However, prominent chemical gradients were observed over the build plate correlated with the different particle sizes in the powder blend [9]. An effect of the particle size on the powder application to the powder bed has been described by other researchers as well, who confirm that small particles are preferably placed at the beginning of the raking process [10,11,12]. This effect results in the need for a feedstock preparation technique that eliminates particle size related segregation effects in the powder blend.

Satelliting is a recently introduced method to enhance adhesion of small particles to large particles. The studies published so far use polyvinyl alcohol (PVA) as the bonding medium between the particles [13,14,15,16]. The present study introduces a pectin binder as a biobased alternative to PVA for the satelliting method and the method is applied to a steel powder with carbide additions for the first time. Small Cr_3_C_2_ carbides with an average size of 5 µm are bonded to larger AISI H13 steel particles (average size of 45 µm) via pectin and processed by PBF-LB/M. The same alloy was prepared via PVA binder as a benchmark.

### 1.2. Polymer Binders: Polyvinyl Alcohol versus Pectin

PVA is a synthetic polymer synthesized by the hydrolysis of polyvinyl acetate and is frequently used in a variety of applications, ranging from adhesives to applications in the textile, medical and food industries [17,18,19,20]. PVA shows good emulsifying and film forming properties, and is commonly selected based on its good adhesion properties [21,22]. Especially in metal binder jetting (MBJ), where metal powder and polymeric binder is printed layer-wise to form a green body, PVA has gained a lot of interest. In contrast to PBF-LB/M, the process requires additional steps for debinding and sintering of the metal powder. PVA and other polymer-based binders are often preferred as they show easy and efficient debinding and work on a variety of materials [23,24]. Different types of PVA are commercially available, mainly differing in their molecular weight and their degree of hydrolysis. However, due to its synthetic origin, a biobased and renewable alternative is desirable.

Pectin is a naturally occurring polysaccharide that recently found its application as a biobased, degradable and environmentally friendly material in medical and biotechnological applications, commonly as a thickening agent [17,25,26]. Pectin is found in the middle lamella and cell walls of several plants and is mostly extracted from citrus-peel and apple pomace, both as a by-product from juice manufacturing [17]. The most important structural motif of pectin is homogalacturonan, made of α-(1,4)-linked D-galacturonic acid repeating units. Different types of pectin are commercially available, differing in molecular weight and their rhamnogalacturonan II regions carrying several different sugars grafted to the backbone [17]. Both PVA and pectin have shown good capabilities in coordinating to metals for the removal of heavy metals in wastewater treatment which is attributable to their functional groups [27,28].

**Figure 1:**
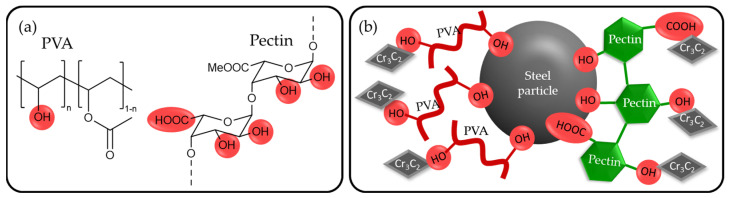
(**a**) Chemical structure of polyvinyl alcohol [20] offering several hydroxy groups, depending on the degree of hydrolysis (**left**) and simplified chemical structure of pectin [30], showing hydroxy- and carboxylic acid groups as possible coordination sites (**right**). (**b**) Conceptualized coordination of functional groups of each binder towards the steel and the carbide particles [31].

Another characteristic of pectin is the degree of methyl esterification of the carboxylic acid groups, defining its gel-forming properties [29]. Therefore, a common classification defines pectins with a degree of methyl esterification of <50% as low-methoxy (LM) pectins [19]. For the application in this work, a low-methoxy pectin is expected to show better coordination to the metals via the carboxylic acid sites. The chemical structure of PVA and pectin is shown in Figure 1a. The conceptualized coordination of the binder’s functional groups towards the powder particles is schematically shown in Figure 1b.

The analyzed alloy is based on spherical, PBF-LB/M suited AISI H13 and modified by additions of 5.0 wt.% edged Cr_3_C_2_. Since the size of the Cr_3_C_2_ particles varies significantly from the size of AISI H13 particles, the feedstock needs to be prepared accordingly. For direct comparison PVA and pectin were used for the satelliting. The effect of the different polymer binders on the powder feedstock and its processability are subject of this study. The feedstocks were characterized in detail by secondary electron microscopy (SEM), in combination with energy dispersive X-ray spectroscopy (SEM-EDS), Fourier-transform infrared spectroscopy (FTIR) and thermogravimetric analysis (TGA)-FTIR. In-depth microstructure analyses of built-up samples are carried out to characterize the effect of the binder-assisted additivation of Cr_3_C_2_ on the tool steel.

## 2. Materials and Methods

### 2.1. Materials

The alloy is based on AISI H13 (Oerlikon MetcoAdd, Raunheim, Germany, AISI H13, X40CrMoV 5-1-1, 45 ± 15 µm) and was modified by additions of 5.0 wt.% Cr_3_C_2_ (5 µm, Höganäs). The spherical AISI H13 powder was gas atomized under nitrogen atmosphere, whereas the Cr_3_C_2_ powder was mechanically crushed and showed an edged shape.

The alloying content of the powders and the composition calculated from the mixing ratio are listed in Table 1.

PVA (M_w_ = 9–10 kDa, 80% hydrolyzed, Sigma Aldrich, St. Louis, MO, USA), pectin (Herbstreith & Fox, Elmsford, NY, USA, Citrus Pectin Classic: CU-901, M_w_ = 9.6 kDa, degree of esterification: 6.6%, galacturonic acid content: 96%), Hellmanex^®^ III solution (VWR), ethanol (Sigma Aldrich, 99.8%, p.a.) and water (Carl Roth ROTISOLV^®^, Karlsruhe, Germany, High-Performance Liquid Chromatography (HPLC) grade) were used without further purification for the satelliting procedure.

### 2.2. Powder Feedstock Preparation

The satelliting was carried out as follows: First, 25.0 g of the corresponding binder (2 wt.%, pectin or PVA) was dissolved in 1000 mL deionized water at room temperature. Afterwards, 1162.5 g steel powder (93 wt.%) and 62.5 g carbide powder (5 wt.%) were added to the solution. The suspension was mixed for 20 min before being spray dried (Büchi Spray Dryer B-290) with the following settings: *T*_in_ = 160 °C, *T*_out_ = 75 °C, $p_{N_{2}}$ = 5 bar, ${\dot{V}}_{N_{2}}$ = 473 L/h, ${\dot{V}}_{feed}$ = 1 L/h. The feedstock powder was obtained in a yield of 65%.

### 2.3. Binder and Feedstock Analysis

#### 2.3.1. Quartz-Crystal Microbalance with Dissipation (QCM-D)

QCM-D was used to measure the ad-/desorption kinetics of the binder on the powder materials. Adsorption describes the binder’s ability to wet the surface of the base material. Desorption is the residue-free removal of the binder when rinsed with water. Therefore, the measurements further reveal whether the adsorption process is reversible. The measurements were performed on a QSense Explorer from Biolin Scientific (Gothenburg, Sweden), equipped with a Flow Module QFM 401 in combination with an Ismatec IPC multichannel peristaltic pump on the prepared feedstock. The simultaneous measurement of the frequency *f* and the dissipation *D* was carried out for the fundamental resonance frequency (*n* = 1, i.e., *f* = 5 MHz) and the six overtones (*n* = 3, 5, 7, 9, 11, and 13 corresponding to *f* = 15, 25, 35, 45, 55, and 65 MHz, respectively) at a flowrate of 50 µL/min and a temperature of 25 °C. The resolution in *f* and *D* is ±1 × 10^−2^ Hz and ±1 × 10^−2^ ppm, respectively. HPLC water was used as a solvent for all experiments. The custom sensors (AISI H13 steel and chromium carbide) were acquired from QuantumDesign Europe (Darmstadt, Germany). The composition of the sensors was determined by XPS (Figure S4). Prior to use, the sensors were cleaned via ultrasonication in Hellmanex^®^ III (Hellma GmbH & Co. KG, Müllheim, Germany) solution (3% (*v*/*v*)) for 10 min, followed by ultrasonication in HPLC water for 10 min and ultrasonication in ethanol. The sensors were dried in a slow N_2_ stream.

In the first phase of the measurement, the custom sensor (steel or chromium carbide) was equilibrated in HPLC water for 20 min. The experiment was then started by exchanging the water with the corresponding solution of pectin or PVA (25 mg/mL). When Δf and ΔD reached a constant value, the polymer solution was exchanged by water to monitor the desorption of the corresponding polymer from the sensor. As soon as no more desorption was observed, the measurement was finished. When plotting the data, the frequency and dissipation data were presented as the difference, $\Delta f_{n}/n$ and ΔD, with respect to their initial value at the beginning of the measurement.

#### 2.3.2. Attenuated Total Reflection Fourier Transform Infrared Spectroscopy (ATR-FTIR)

Infrared spectra were obtained to characterize the feedstock powders using a Perkin Elmer (Waltham, MA, USA) Spectrum 3 FTIR spectrometer with a GladiATR diamond crystal. These spectra qualitatively prove that the binder is retained within the feedstock after the spray drying procedure. The measurements were performed at wavenumbers from 4000 cm^−1^ to 400 cm^−1^ with a resolution of 4 cm^−1^ and 4 scans per measurement at room temperature. All spectra were baseline corrected and normalized with Spectrum 10, PerkinElmer and processed via OriginPro 2018, OriginLab.

#### 2.3.3. Thermogravimetric Analysis (TGA)

Thermogravimetric analysis was used to characterize the thermal decomposition behavior for the two binders and quantify the binder content within the pectin and PVA-bonded-feedstocks. The analyses were performed on a STA 6000 Thermal Analyzer by PerkinElmer coupled to a gas-phase FTIR spectrometer under nitrogen atmosphere for the two binders and argon atmosphere for the satellited feedstocks to prevent nitride formation. The measurements were performed from 30–950 °C at a rate of 20 °C/min using ~30 mg of sample. The DTG (Derivative thermogravimetry) curve was generated by differentiation of the measured TGA curve using OriginPro 2018 software. The decomposition temperature was identified as the minimum of the DTG curve, and the binder content was calculated as the difference between the sample mass at 100 °C and 950 °C to account for the loss of moisture below 100 °C.

#### 2.3.4. Scanning Electron Microscopy—Energy-Dispersive X-ray Spectroscopy (SEM-EDS)

SEM-EDS elemental distribution mappings of the feedstocks were recorded on Hitachi (Tokyo, Japan) SU9000 electron microscope at a beam voltage of 15 kV.

### 2.4. Powder Application and PBF-LB/M Processing

Powder application tests and PBF-LB/M processing were performed with a Realizer SLM 100 (Realizer GmbH, Borchen, Germany) machine. The application tests were conducted at room temperature and 500 °C to evaluate the feedstocks’ behavior.

The specimens for microstructural analysis and hardness testing were processed with a pulsed ytterbium fiber laser (1070 ± 2.5 nm) and 500 °C substrate plate heating. The exposure point distance of 50 µm and exposure time of 60 µs result in the scanning speed v of 833 mms^−1^. A laser power P of 160 W, hatch distance h of 0.1 mm and layer thickness d of 30 µm were used. The energy density E_v_, provided in Equation (1), was used to describe the irradiated laser energy in a specific volume element, and was equal to 64 J mm^−3^.

(1) $$E_{v}=\frac{P}{h\cdot v\cdot d}$$

A stripe scanning strategy with a width of 2.7 mm was applied, leading to an overlap of 0.1 mm to the sides of each stripe. Within the stripes, the exposure was carried out in a zig-zag pattern. The hatch direction was rotated by 90° after each layer. Argon was used as process gas. Cooling from the process temperature to 60 °C takes approximately 60 min. The process chamber was constantly kept under argon atmosphere during cooling.

### 2.5. Microstructural Analysis

The specimen was cut vertically, and the surface was ground and mechanically polished to 0.05 µm. The specimens were polished electrolytically with A2 electrolyte after grinding and mechanical polishing steps to eliminate any residual surface deformation for the subsequent electron backscatter diffraction (EBSD) measurements.

Backscatter electron images (BSI), as well as coupled EBSD and SEM-EDS measurements were recorded with a Helios Nano Lab Dual Beam, (Thermo Fisher Scientific, Hillsboro, OR, USA) and parameters were set to 15 kV and 5.5 nA.

Coupled EBSD and SEM-EDS measurements enable a characterization of the phase distribution in relation to the solidification related segregations. The scans were recorded with TSL OIM Data Collection 7 (EDAX, AMETEK, Berwyn, IL, USA). A step size of 50 nm was chosen. Fcc (a = 3.65 Å) and bcc (a = 2.87 Å) crystal structures were considered in the measurements. Before the measurement, a calibration of the software to the present microstructure was performed. Inverse pole figure (IPF) maps are used for qualitative texture analysis of the material’s matrix. The texture analysis was performed with OIM Analysis 7 (EDAX, AMETEK, Berwyn, IL, USA) software. Points with a confidence index < 0.1 are removed by a clean-up procedure.

## 3. Results

### 3.1. QCM-D Reveals That Pectin Irreversibly Adsorbs to Steel, in Contrast to PVA

**Figure 2:**
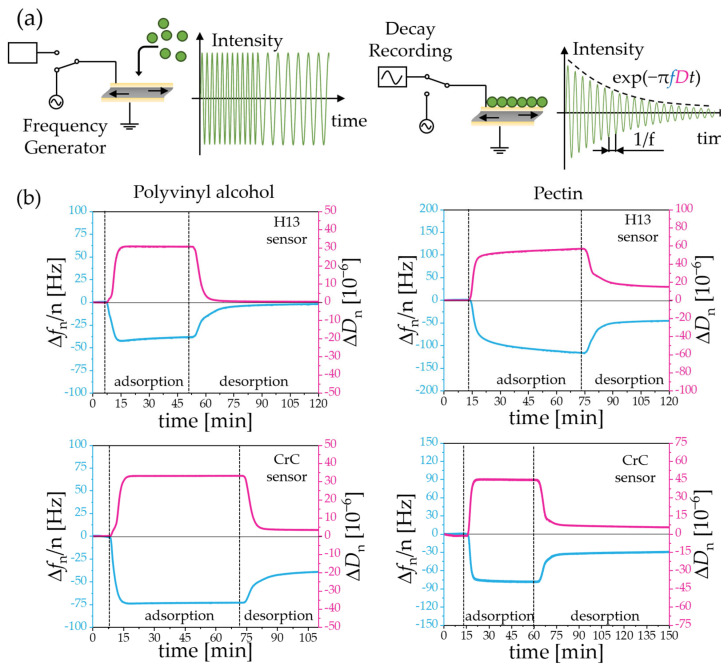
(**a**) QCM-D principle: Excitation of a resonating quartz crystal, followed by recording of the frequency change Δ*f* and dissipation change Δ*D* as a measure of energy loss per cycle. A mass uptake on the surface leads to a decrease in frequency, while the dissipation increases for the adsorption of viscous films. (**b**) Real-time changes in the frequency $\Delta f_{n}$ and the dissipation $\Delta D_{n}$ by QCM-D at 25 °C for polyvinyl alcohol and pectin using sensor-surface materials of AISI H13 tool steel (**top**) and chromium carbide (**bottom**). The third overtone (*n* = 3) is shown representatively [34]. Each measurement consists of three phases: equilibration in water, adsorption with a binder solution (2.5% (*w*/*v*)), and desorption via rinsing with water, marked by the dashed lines.

QCM-D experiments were carried out to provide a direct comparison of the adsorption capabilities of both binder candidates. In QCM-D measurements, a mass change (e.g., 17.7 ng/[cm^2^ Hz] for a 5 MHz quartz crystal [33]) on the surface was measured by the shift in the resonance frequency of the quartz crystal. A schematic drawing of the operating principle is shown in Figure 2a. Adsorption leads to a higher areal mass density, shifting the resonance frequency to lower values. When the excitation of the sensor was stopped, a decay of the signal was recorded, correlating with the dissipation D, which provides information about the softness of the adsorbed layer (Figure 2a). For each of the two binder solutions, PVA and pectin, the experiments were performed for each on a steel and chromium carbide sensor.

The dependency of the changes in frequency Δf and dissiation ΔD on time are shown in Figure 2b for PVA and pectin on steel and chromium carbide sensors, respectively. After changing the solvent to the binder solution, Δf drops to lower values due to an increase in viscosity of the solution as well as adsorption of the binder to the sensor. An equilibration state is reached after less than 15 min for the adsorption of PVA on steel and carbide as well as pectin on the carbide. This observation indicates the completion of binder adsorption on the metal surface. The adsorption of pectin on the steel surface is slower as the equilibration state is not reached even after 1 h of using the binder solution as shown by the ongoing increase in Δf. As the greatest increase takes place in the first 15 min, the following feedstock preparation will only be conducted for 15 min to avoid corrosion of the steel. When the binder solution is exchanged by pure water again, desorption is taking place within around 15 min until Δf remains at a constant value, not going back to its original value except for PVA at the H13 steel surface. Therefore, PVA adsorption on steel seems to be completely reversible. In contrast to this, pectin shows a lasting surface coating for both types of surface, thus making it better suited for the satelliting process. A quantitative comparison of the adsorption is not possible because of the different viscoelastic properties of both polymer solutions and the material constant of custom sensors is unknown.

### 3.2. Spray Drying Yields Steel Powder Satellited with Chromium Carbide by PVA or Pectin

**Figure 3:**
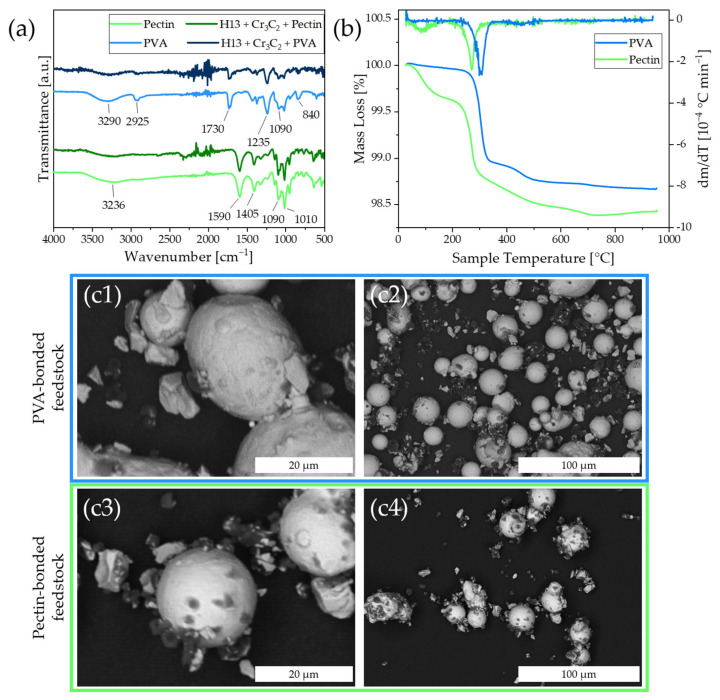
(**a**) FTIR spectra of the binders pectin and PVA in their pure state compared the corresponding powder feedstocks consisting of 2% (*m*_binder_/*m*_feedstock_) binder, 5% (*m*_carbide_/*m*_feedstock_) chromium carbide and 93% (*m*_steel_/*m*_feedstock_) H13 steel obtained from spray drying. (**b**) TGA and DTG curve of the two powder feedstocks. (**c1**–**c4**) SEM images show the localization of the binder and carbide on the steel surface (BSE, 15 kV).

According to previous results, the suspension was mixed for 15 min before spray drying to guarantee the adsorption of binder on the steel and carbides. The binder which is dissolved in the solvent absorbs to the powder surface during spray drying. The measured FT-IR spectra of the spray dried feedstocks qualitatively confirm the presence of the binder in the feedstock as all characteristic IR absorption bands of the corresponding binder are also observed for the feedstock (Figure 3a).

The thermogravimetric analysis of the two powder feedstocks (Figure 3b) shows a thermal decomposition of the organic material. The main decomposition temperatures correlate with the minima at the DTG curve (Figure 3b) and are 270 °C for pectin and 305 °C for PVA. The corresponding FT-IR spectra of the gaseous decomposition products (Figure S1) show an intense signal for the pectin-bound alloy at a temperature of 260 °C, which is correlated with the release of CO_2_ but slightly shifted in terms of temperature values due to the flow of the decomposition products to the FT-IR gas analyzer. The PVA sample, in contrast, does not show any intense IR-signals as seen in the IR-intensity profile in Figure S1 at its main decomposition temperature, which indicates less volatile decomposition products in the powder formulation. As water evaporates below 100 °C the binder content is calculated from the weight loss from 100 °C up to 950 °C, resulting in 1.3% (*m*_binder_/*m*_feedstock_) and 1.4% (*m*_binder_/*m*_feedstock_) for PVA and pectin, respectively.

The results for the thermal decomposition of the pure binders obtained from the thermogravimetric analysis are provided in Figure S3.

Scanning electron microscopy was used for the visualization of the additivated chromium carbide and the binder on the steel particles (Figure 3c). The SEM images show the adhesion of smaller carbide particles on the surface of large steel granules for both feedstocks.

At the surface of the steel particles, spherical and mushroom-cap-shaped pectin particles are visible and can be seen as dark particles in the BSE-image (Figure 3c) and were confirmed by SEM-EDS-measurements (Figure S2), while PVA showed less of those particles. Walton et al. reported that spray drying of polymers can yield different particle morphologies depending on the process temperatures, ranging from spherical particles to mushroom-cap-shaped and raisin-like appearance [35,36].

As many carbides are located at the surface of the steel particles where no binder is visible, for PVA, it is expected that a thin film, deposited on the surface of the steel and carbide particles, is responsible for the successful adhesion of both, carbides, and steel. For pectin, more agglomerates were found as spherical and mushroom-cap shaped particles (Figure S2). The reduction of the pectin concentration in future experiments may reduce the formation of polymer agglomerates and lead to more homogeneous coatings of powders.

### 3.3. Powder Application and PBF-LB/M Processing

**Figure 4:**
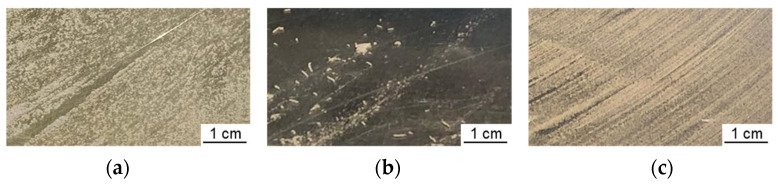
Substrate plates with 1 powder layer: (**a**) PVA-bonded alloy applied at 20 °C, (**b**) PVA-bonded alloy applied at 500 °C and (**c**) pectin-bonded alloy applied at 500 °C.

Images of the substrate plate after raking the first powder layer are shown in Figure 4. The PVA-bonded alloy can be applied on a substrate plate at room temperature. Slight agglomeration can be observed. At 500 °C, the PVA-bonded alloy could not be applied onto the substrate plate. The feedstock shows a strong tendency to form agglomerates, and a homogeneous application of the feedstock is impossible. The pectin-bonded alloy can be applied onto the substrate plate at 500 °C, and no agglomeration is observed. The raking direction within the applied powder layer is visible in all three images.

**Figure 5:**
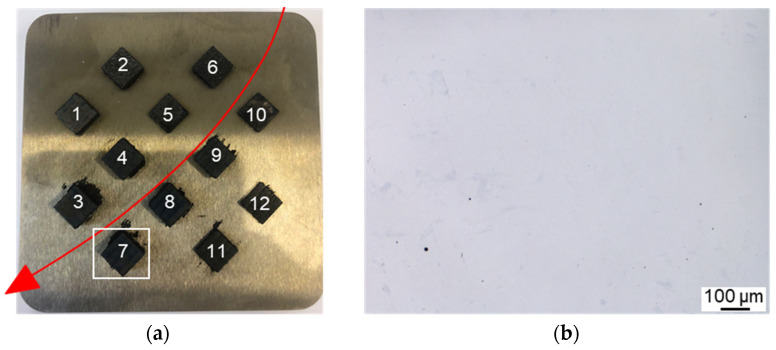
(**a**) Substrate plate with 12 built-up specimens, the powder application direction is indicated by the red arrow. (**b**) Unetched LOM image of the microstructure in specimen 7, revealing high density and no cracks.

Since the PVA-bonded alloy could not be applied to the substrate plate at elevated temperatures, only the pectin-bonded alloy was processed. The built-up specimens are depicted in Figure 5a. The specimens appear dark grey instead of the usual metallic silver. The oxygen content of the specimens was determined to be 978 ± 221 ppm.

Specimen 7 showed the best relative density without hot cracks, and was therefore analyzed in more detail. Furthermore, results from reference specimens without binder are available [9] and a comparison to the processing of the same alloy with identical processing parameters without a polymer binder was possible. The relative density of the specimen was >99.9%. Some small pores are evident within the microstructure in Figure 5b.

Optical emission spectroscopy (OES) measurements of specimen 7 of the pectin-bonded alloy compared to the identically processed specimen 7 without binder are provided in Table 2. The Cr and C contents of the alloy containing the binder were higher, suggesting a higher fraction of carbides in the specimen. Without binder, the chemical gradients across the substrate plate were significant. A variation between 7.1–8.5% Cr was observed within the non-satellited sample set [9]. The variation within the satellited feedstock was lower; six samples were measured, and Cr-contents varied between 8.9–9.7% (average of 9.2%). Specimen 7 had a Cr content of 9.3, which is close to the expected calculated composition. The measured Cr contents of the specimens from the satellited feedstock lay closer to the ideal composition.

### 3.4. Microstructural Analysis

In order to evaluate the influence of the satelliting process on the microstructure, two specimens of almost identical composition and identical processing parameters were compared: Specimen 7 of the pectin-bonded alloy and specimen 7 of a simple powder blend of AISI H13 with 13 wt.% Cr_3_C_2_ in the feedstock, of which only a smaller fraction was integrated into the specimen. This specimen was previously calculated to contain 5.4 wt.% of Cr_3_C_2_. The rest of the Cr_3_C_2_ is lost during the raking process due to segregations phenomena in the powder blend [9]. The compositions of the specimens to be compared are listed in Table 3.

**Figure 6:**
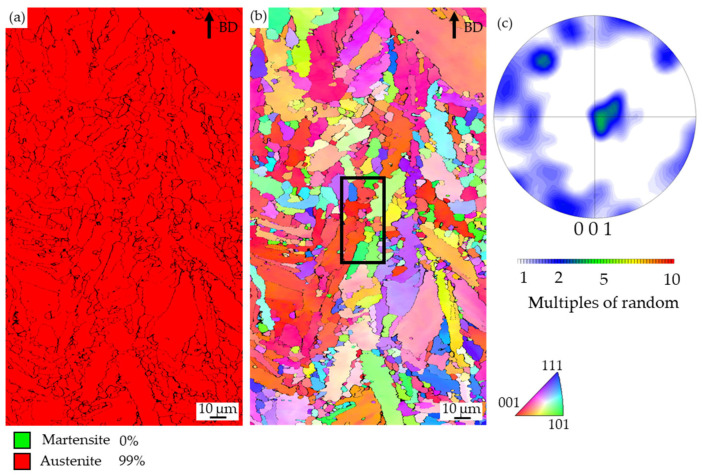
(**a**) Phase analysis, (**b**) IPF map, and (**c**) corresponding pole figure of specimen 7 of the pectin-bonded alloy with 5 wt.% of Cr_3_C_2_. In (**b**), the area subjected to further, detailed analysis is marked by the black box.

The EBSD measurements revealed the microstructure of specimen 7 of the pectin-bonded alloy to be fully austenitic, as shown in Figure 6a. The reference specimen showed a martensite content of 43 vol.% [9].

The grains, easily distinguishable from the IPF map in Figure 6b, show an inhomogeneous grain size. Some columnar grains are observed which have grown over several processed layers (d = 30 µm). The pole figure shows a preferred orientation of the grains. However, many small grains are also present, and the observed texture is lower than was previously observed in pure AISI H13 [37]. Within the large grains, e.g., in the upper right corner, different shades in the color coding indicate some orientation differences within the grains, possibly resulting from distortions. Distortion within the austenite grains can result from the dendritic solidification within the grains accompanied by slight chemical segregations. To analyze distortions in more detail, the grain boundary orientations were evaluated. A fraction of 30.3% of grain boundaries were low-angle grain boundaries < 15°, whereas 69.7% were high-angle grain boundaries. Low-angle grain boundaries are often associated with an increased dislocation density resulting in the misorientation between the (sub)grains.

**Figure 7:**
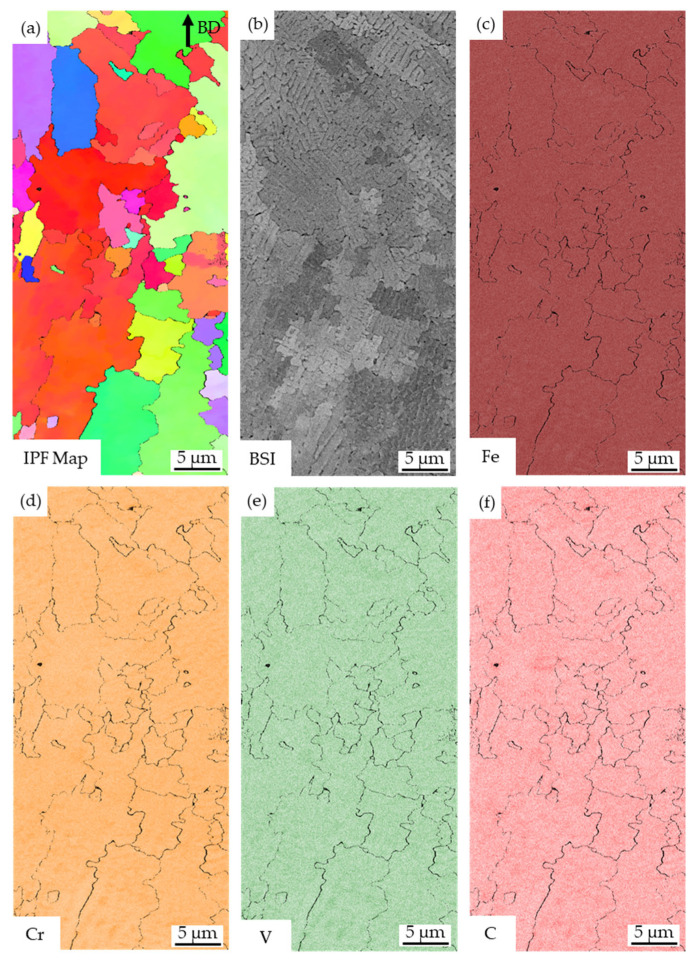
(**a**) IPF map of a smaller section within the microstructure, (**b**) the correlating BSI. (**c**) The Fe map, (**d**) Cr map, (**e**) V map, and (**f**) C map from an EDS scan reveal a homogenous chemical composition with slight segregations of Cr, V, C in interdendritic regions.

A more detailed analysis of the marked area in Figure 6 is shown in Figure 7. The IPF map is correlated with a BSI (Figure 7b). The contrast in the BSI mainly results from the differences in the orientation of the grains. EDS maps of the microstructure revealed a very homogenous distribution of the alloying elements. No areas were found, in which Cr and C were significantly enriched. Slight segregations related to the solidification of the dendrites were apparent (e.g., in the bottom grains). The carbide-forming elements, Cr, V, C were enriched in the interdendritic regions, whereas slightly less Fe appeared in these areas.

## 4. Discussion

### 4.1. Powder Feedstock—Preparation and Characteristics

The time-resolved adsorption of the two binders was studied via QCM-D, revealing the effective binding of pectin on both steel, and chromium carbide. The fabrication of a satellited feedstock was successfully achieved by spray drying of an aqueous binder solution together with the steel powder and the additivated carbides. The spray drying process offers great opportunities with regard to up-scaling for the fabrication of larger quantities. The fact that free binder granules are found in the formulation, which are not bonded to steel or carbide particles, indicates that the binder content could be further reduced. The thermal decomposition of both binders was studied via TGA and showed that PVA has a higher decomposition temperature than pectin. Nevertheless, it is the melting of the synthetic polymer that holds back its capabilities to be applied in PBF-LB/M as it drastically impairs the flowability and raking behavior.

### 4.2. Powder Application and PBF-LB/M Processing

The powder properties and their applicability to the substrate plate are temperature dependent. At elevated temperatures, the PVA-bonded alloy showed significantly reduced flowability and strong agglomeration. This might be caused by melting of PVA compared to pectin, which undergoes multiple decomposition reactions at elevated temperatures [38,39]. While PBF-LB/M processing of the PVA-bonded alloy was impossible due to the decreased rakeability, the powder bed of the pectin-bonded alloy was sufficient for subsequent exposure by the laser at elevated temperatures.

Although specimen 7 lay at the end of the raking length, the composition in the specimen fit well with the expected composition of an ideally mixed alloy. Specimen 7 without binder showed the largest deviation from the ideal composition [9]. These comparisons suggest that the raking process with the satellite feedstock was significantly more stable, and the chemical gradients could be reduced by using the satelliting technique for the feedstock preparation. The elevated base plate temperature does not negatively affect the bonding of the powder during the application of the powder. However, the decomposition of the pectin identified in the FTIR spectra is also expected to occur after the application of the powder onto the powder bed. The decomposition of the binder at elevated temperatures is most likely the reason for the enhanced carbon content that was observed in the satellited feedstock compared to the simple powder blend.

### 4.3. Microstructure Analysis

The PBF-LB/M processing parameters used were well-suited to processing the satellited feedstock and produced samples with low porosity. Cracks are known to appear in the microstructure when processing tool steels, but none were apparent in this study. Furthermore, the chemical homogeneity within the microstructure of the specimens was high. The Cr_3_C_2_ particles were fully molten, and the convection within the melt pool resulted in a stable in situ alloying process. If the energy input was too low, the convection would be insufficient for complete homogenization of the melt and microstructural gradients would appear and be made visible by the EDS measurements.

The three compositions in Table 3 are very similar, but the resulting microstructure varies significantly in the content of the retained austenite content. Carbon has the largest impact on the martensite transformation temperature in this alloying system. Small variations in C content can impact the austenite content to a large extent [40]. Compared to the reference specimen without binder, the microstructure of the pectin-bonded alloy shows significantly more austenite. Interstitial carbon within the lattice structure of the austenite enhances its stability. The carbon content of the pectin-bonded specimen is higher compared to the specimen derived from a simple powder blend. As pectin is an organic binder, the enrichment in carbon is possibly related to residual binder being integrated into the microstructure during the PBF-LB processing of the satellited powder feedstock. The additional carbon in these concentrations does not negatively influence the processability of the alloy in the chosen PBF-LB/M parameters yet. However, the binder content should be monitored and preferably reduced to influence the attained microstructure even less. A hardening heat treatment for the reference specimen has proven to be sufficient to transform the large austenite content to martensite. A verification will be made for the present alloy in the future. A slightly increased carbon content might be acceptable in the alloy if hardenability and processability are maintained. The carbon might even positively influence the hardness and strength of the tool steel alloy.

## 5. Conclusions

A powder blend of AISI H13 was additivated with 5 wt.% of Cr_3_C_2_ with pectin as a binder to enhance adhesion of small Cr_3_C_2_ particles to AISI H13 steel particles. From the results of this research, the following conclusions can be drawn:

- The potential of pectin as a biobased binder for the satelliting process was shown. It adsorbs on the surface of the steel and carbide and enhances the adhesion of small carbide particles to larger steel particles.
- The PVA-bonded alloy as a benchmark for the satelliting process shows significantly reduced processability at pre-heating temperatures of the substrate plate of 500 °C.
- The pectin-bonded alloy could be applied at 500 °C and specimens were built-up from the pectin-bonded alloy.
- The chemical gradients over the substrate plate related to the particle size can be reduced by using a by pectin satellited feedstock compared to a simple powder blend. The adhesion of the carbide particles to the steel particles via pectin is stable enough to endure the mechanical forces of the recoating unit on the feedstock.
- The carbon content within the specimens of the satellited feedstock is enhanced, possibly being a resulting effect of the organic nature of the binder. The binder content in this initial study is 2 wt.% and will be reduced in future studies. The effect of the carbon enrichment will be analyzed carefully.
- The processing parameters used provide adequate mixing of all alloying components in the melt pool. No cracks were found, the porosity lay well below 0.1% and the chemical composition was homogenous, and no C or Cr enrichments related to an inhomogeneous Cr_3_C_2_ distribution were found.

## Figures and Tables

**Table 1:** Nominal composition of the mixing components, calculated theoretical composition of the mixed powder [wt.%].

Name	C	Si	Mn	Cr	Mo	V	Fe
AISIH13 nominal [32]	0.35–0.42	0.8–1.2	0.25–0.5	4.8–5.5	1.2–1.5	0.85–1.15	Bal.
AISI H13 as provided	0.37	0.9	0.4	5.0	1.3	1.1	Bal.
Cr_3_C_2_ nominal	13.3	-	-	86.7	-	-	-
H13 + 5% Cr_3_C_2_ calculated (ideal)	1.1	0.9	0.5	9.4	1.2	1.0	86.0

**Table 2:** Chemical composition of specimen 7, processed with 64 J/mm^3^, determined by OES of the pectin-bonded alloy and a regular powder blend of AISI H13 with 5 wt.% Cr_3_C_2_.

Name	E_V_	C	Si	Mn	Cr	Mo	V	Fe
	[J/mm^3^]	[wt.%]	[wt.%]	[wt.%]	[wt.%]	[wt.%]	[wt.%]	[wt.%]
Calculated AISI H13 + 5 wt.% Cr_3_C_2_		1.1	0.9	0.5	9.4	1.2	1.0	86.0
Specimen 7 AISI H13 + 5 wt.% Cr_3_C_2_	64	0.6	0.7	0.3	7.1	1.4	1.0	88.0
Specimen 7 Pectin-bonded alloy with 5 wt.% Cr_3_C_2_	64	1.2	0.8	0.3	9.3	1.3	0.9	85.5

**Table 3:** Comparison of the chemical composition of the two specimens for microstructure analysis with and without binder in the feedstock and comparison to the calculated ideal composition.

Name	E_V_	C	Si	Mn	Cr	Mo	V	Fe
	[J/mm^3^]	[wt.%]	[wt.%]	[wt.%]	[wt.%]	[wt.%]	[wt.%]	[wt.%]
Calculated AISI H13 + 5 wt.% Cr_3_C_2_		1.1	0.9	0.5	9.4	1.2	1.0	86.0
Specimen 7 Pectin-bonded alloy with 5 wt.% Cr_3_C_2_	64	1.2	0.8	0.3	9.3	1.3	0.9	85.5
Specimen 7 AISI H13 + 13 wt.% Cr_3_C_2_	64	1.0	0.8	0.3	9.7	1.3	1.0	85.2

## Data Availability

Data and materials are available upon request from the authors.

## Supplementary Materials

The following supporting information can be downloaded at: https://www.mdpi.com/article/10.3390/ma16103649/s1, Figure S1: FT-IR spectra of pyrolysis gas for (a) pectin and (b) PVA-bonded alloy, showing the volatile decomposition products evolved during TGA. The spectra were recorded from 4000–400 cm^−1^ at a resolution of 4 cm^−1^. TGA was performed at a heating rate of 20 °C/min under argon atmosphere (Ar-flow: 150 mL/min); Figure S2: (a) SEM image: Morphology of mushroom-cap shaped pectin particles on the surface of chromium carbide particle, attached to a larger steel particle (SEM, Hitachi SU9000, SE, 30 kV) (b) SEM-EDX image of a single steel granule, decorated with Cr_3_C_2_ particles. (Hitachi SU9000); Figure S3: (a) TGA analysis of the pure binders PVA and pectin and (b) the corresponding FTIR data of the pyrolysis gases of PVA and (c) for pectin; Figure S4: XPS-analysis survey of the sensor surfaces and chemical composition for the steel sensor (left) and the chromium carbide sensor (right).
