# Supplementary material for: Novel Pectin Binder for Satelliting Carbides to H13 Tool Steel for PBF-LB Processing
Source: Materials (Basel). 2023 May 10;16(10):3649. doi: 10.3390/ma16103649 (PMC10222759; doi:10.3390/ma16103649)
Supplement: Supplementary file 1 [file materials-16-03649-s001.zip › materials-2367867-supplementary.pdf]

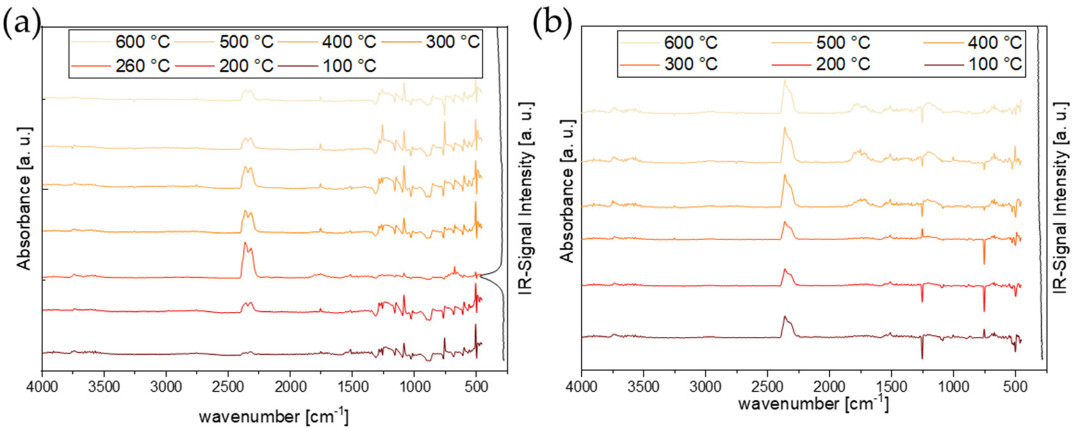

Figure S1. FT-IR spectra of pyrolysis gas for a) pectin and b) PVA-bonded alloy, showing the volatile decomposition products evolved during TGA. The spectra were recorded from 4000-400 cm^{-1} at a resolution of 4 cm^{-1}. TGA was performed at a heating rate of 20 °C/min under argon atmosphere (Ar-flow: 150 mL/min)

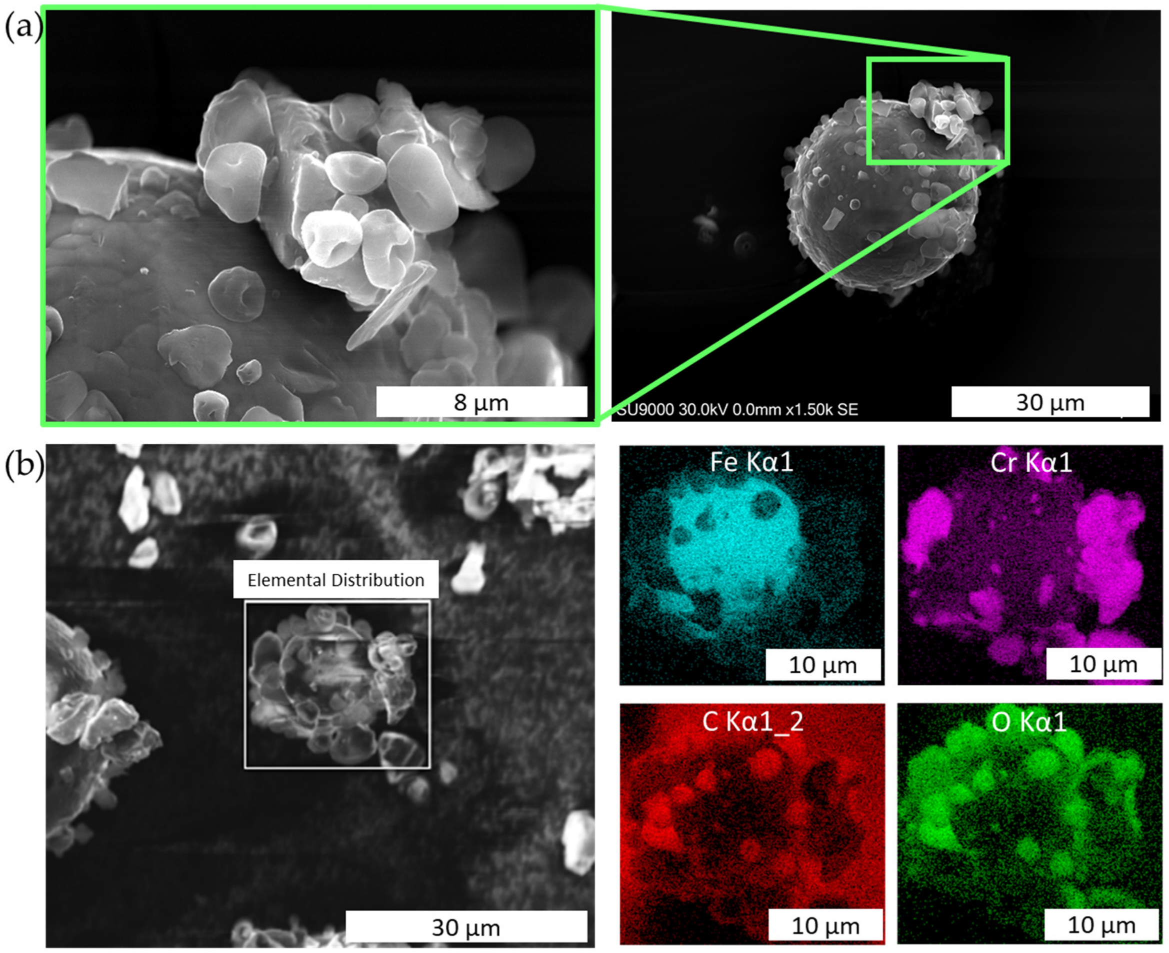

Figure S2. a) SEM image: Morphology of mushroom-cap shaped pectin particles on the surface of chromium carbide particle, attached to a larger steel particle (SEM, Hitachi SU9000, SE, 30 kV) b) SEM-EDX image of a single steel granule, decorated with Cr_3C_2 particles. (Hitachi SU9000)

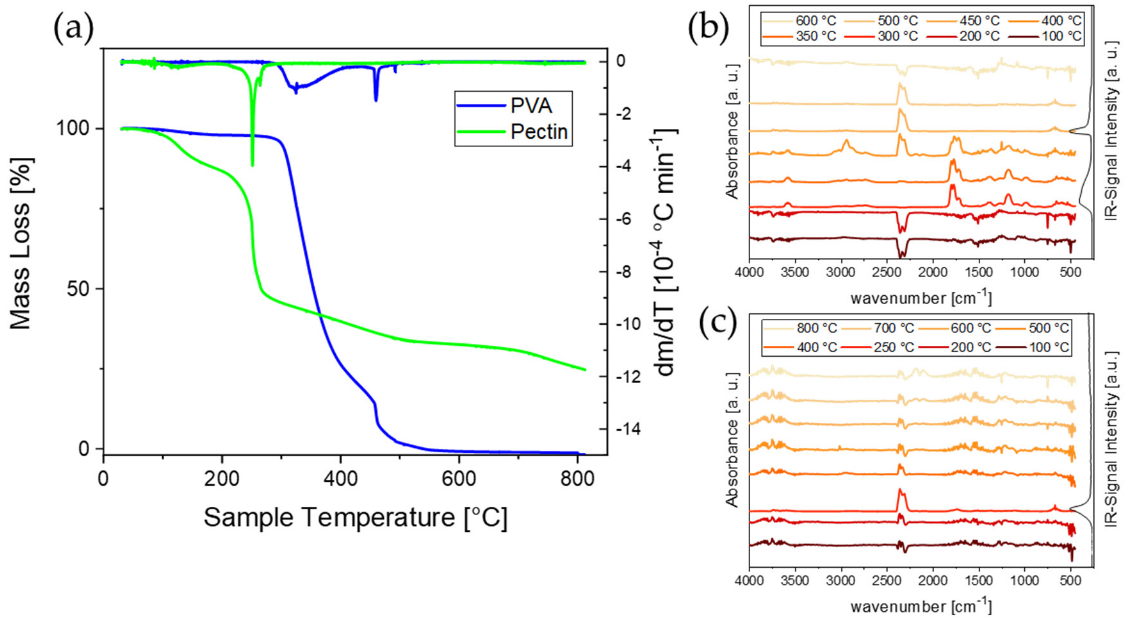

Figure S3. (a) TGA analysis of the pure binders PVA and pectin and (b) the corresponding FTIR data of the pyrolysis gases of PVA and (c) for pectin.

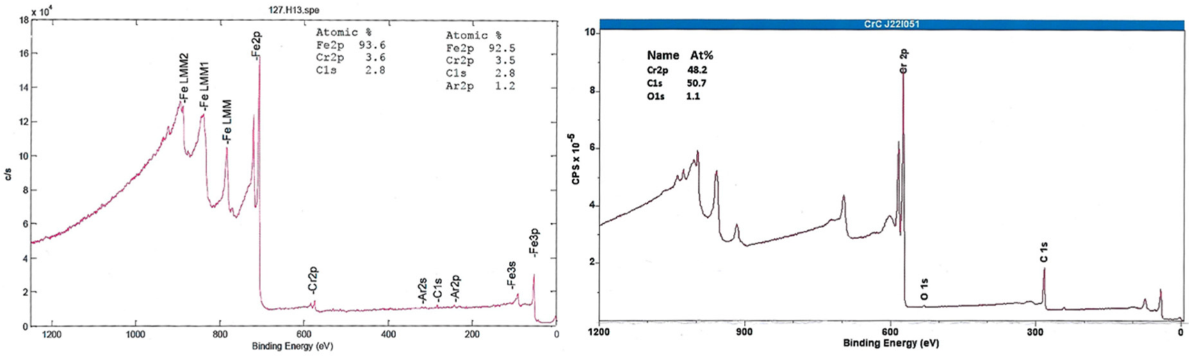

Elements	Atomic %
Fe	93.6
Cr	3.6
C	2.8

Elements	Atomic %
Cr2p	48.2
C1s	50.7
O1s	1.1

Figure S4. XPS-analysis survey of the sensor surfaces and chemical composition for the steel sensor (left) and the chromium carbide sensor (right)
